# Relationship Between Earlobe Crease and Brachial-ankle Pulse Wave Velocity in Non-hypertensive, Non-diabetic Adults in Korea

**DOI:** 10.4178/epih/e2009002

**Published:** 2009-10-12

**Authors:** Sang In Choi, Hee Cheol Kang, Choon Ok Kim, Seung Beom Lee, Won Ju Hwang, Dae Ryong Kang

**Affiliations:** 1 Department of Family Medicine, Yonsei University College of Medicine, Severance Hospital, Seoul, Korea.; 2 Department of Community Health Systems, University of California San Francisco, CA, USA.; 3 Graduate School of Public Health, Yonsei University, Seoul, Korea.

**Keywords:** Association, Earlobe crease, Brachial-ankle pulse wave velocity, Korean

## Abstract

**OBJECTIVES:**

Several studies have found a significant association between the presence of earlobe crease (ELC) and cardiovascular disease (CVD). Brachial-ankle Pulse Wave Velocity (baPWV) is a non-invasive and useful measure of arterial stiffness predicting cardiovascular events and mortality. However, few studies have reported the relationship between ELC and baPWV as a new measure of arterial stiffness. The purpose of this study was to determine whether ELC is related to baPWV in non-diabetic, non-hypertensive, and apparently healthy Korean adults.

**METHODS:**

A cross-sectional study was conducted on 573 non-hypertensive, non-diabetic Korean adults aged 20-80 yr. Subjects were stratified into three groups according to gender and menopausal status. baPWV was measured by an automatic waveform analyser. The association between ELC and baPWV was assessed by multiple linear regression analysis after adjusting for conventional cardiovascular disease risk factors including age, gender, blood pressure, lipid profile, and smoking status etc.

**RESULTS:**

The overall frequency of ELC was 19.02% and the subjects with ELC showed significantly higher mean baPWV (p<0.0001). Multiple linear regression of subjects revealed that the presence of ELC was independently associated with baPWV (male, p<0.0001; premenopausal female p=0.0162; postmenopausal female p=0.0208).

**CONCLUSION:**

ELC had a significant correlation with baPWV, independently controlling for other classical cardiovascular risk factors in adults aged 20 yr or older. ELC is an important surrogate marker of increased arterial stiffness as measured by baPWV in Korean adults.

## INTRODUCTION

The association between diagonal earlobe crease (ELC) and cardiovascular diseases (CVD) was first reported in 1973 [1]. Subsequently, several studies have found a significant association between the presence of ELC and CVD [2, 3]. Moreover, it has been reported [4] that the association between ELC and carotid intima-media thickness (IMT), which is widely used as a surrogate marker for atherosclerotic disease, has been shown to predict the incidence of CVD events [5-7] and has been associated with cardiovascular risk factors [8, 9].

Atherosclerosis is an essential process in the development of major cardiovascular and cerebrovascular events. Characteristic changes of atherosclerosis include stiffness, thickness, and inflammation of arterial walls [10, 11]. Therefore, early detection of functional, structural, and biochemical arterial wall changes may identify patients at high risk of clinical complications from atherosclerosis. Pulse wave velocity (PWV) is the most widely used measure of arterial stiffness in a wide variety of clinical fields [12]. Recently, approaches to measuring arterial stiffness including that of the aorta, which passes over the lower limb arteries, have been of interest [13]. Brachial-ankle PWV (baPWV) is a non-invasive and useful measure of arterial stiffness [14, 15] whose physiological characteristics are closer to those of carotid-femoral PWV than of femoral-ankle PWV [16]. The reproducibility of this measure has been validated [14, 15]. Recent data has demonstrated that higher baPWV is associated with more advanced atherosclerotic changes of the arterial wall in clinical patients [16, 17] and subclinical individuals [18, 19]. However, no previous study has investigated the relationship between the presence of ELC and baPWV. In this cross-sectional study, we examined the association between ELC and baPWV in Korean adults in a health examination programme.

## MATERIALS AND METHODS

### Study subjects

The present study was designed as a cross-sectional study. A total of 852 adults aged 20-80 yr who had a regular physical health examination (including a baPWV measurement) at the Health Promotion Center of Severance Hospital in Seoul from November 2007 to March 2008 were invited to participate. Of the total subjects recruited for the study, 279 were excluded from the analysis; for 32 the diagnosis of ELC was not clear, 16 had self-reported CVD, 149 had hypertension, 28 had diabetes mellitus (DM), 31 did not precisely recall the date of their last menstrual period or did not answer the questionnaire, and 23 had an ankle/brachial pressure index of less than 0.9. In total, 573 subjects were enrolled in the present study. CVD was defined by: 1) self-reported myocardial infarction, angina, or use of nitroglycerin; 2) self-reported history of coronary angioplasty or coronary artery bypass surgery; 3) self-reported stroke, transient ischemic attack, or carotid endarterectomy. Hypertension was defined as systolic blood pressure (SBP) ≥140 mmHg and/or diastolic blood pressure (DBP) ≥90 mmHg or using antihypertensive drugs. DM was defined as fasting blood sugar ≥126 mg/dL and/or use of pharmacological treatment. Hypertensive or diabetic subjects were strictly excluded. The menopausal status of the women involved in this study was confirmed by interview. Women who had not menstruated in 12 months were considered to be menopausal.

The presence of ELC was assigned to subjects with a crease stretching obliquely from the outer ear canal towards the border of the earlobe without discontinuity. Unilateral or bilateral earlobes were all considered to indicate the presence of ELC. Those who have an earring or piercing or revealing incomplete ELC patterns, in which a diagnosis of the ELC might be confused, were not included from the analysis. The study was approved by the Institutional Review Board at Severance Hospital, Yonsei University College of Medicine in Seoul, Korea. Informed consent was received from the subjects.

### Assessment of anthropometric and cardiometabolic factors

While they were wearing light clothes and no shoes, the height and weight of subjects were measured in units of 0.1 cm and 0.1 kg, respectively. Body mass index (BMI) was calculated by dividing weight (kg) by the square of height (m), according to the guidelines recommended by National Institutes of Health. Waist circumference (WC) was measured by the same tester in units of 0.1 cm on the horizontal plane to the surface directly above the iliac crest in the upright position. A sample of fasting venous blood was taken for the measurement of glucose, total cholesterol, high-density lipoprotein (HDL) cholesterol and low-density lipoprotein (LDL) cholesterol, and triglyceride. Serum biochemical parameters were measured after at least 10 hr of fasting. The results from the blood samples were analysed using a Hitachi-7600 (Hitachi High-Technologies Co., Tokyo, Japan).

### Measurement of baPWV

In brief, baPWV was measured using an automatic waveform analyser model BP-203RPE (Colin Co., Komaki, Japan) which was described in detail elsewhere [20]. This instrument simultaneously records blood pressure, phonocardiogram, electrocardiogram, and arterial blood pressure at both the left and right brachial arteries as well as ankles. Subjects were examined in the supine position after 10 min of bed rest. Electrocardiogram electrodes were placed on both wrists and a microphone for the phonogram was placed on the left edge of the sternum. Pneumonic cuffs were wrapped around both the upper arms and ankles and connected to a plethysmographic sensor to determine the volume pulse waveform. Waveforms for the upper arm (brachial artery) and ankle (tibial artery) were stored as 10 sec sample times with automatic gain analysis and quality adjustment. An oscillometric pressure sensor was attached to the cuffs to measure blood pressure at the four extremities. baPWV was recorded using a semiconductor pressure sensor (1,200 Hz sample acquisition frequency) and calculated using the following equation: (*La-Lb*)/*ΔTba*. *La* and *Lb* were defined as the distance from the aortic valve to the elbow and the distance from the aortic valve to the ankle, respectively. The distance from the suprasternal notch to the elbow (*La*) and from the suprasternal notch to the ankle (*Lb*) were expressed as follows: *La*=0.2195*height (cm) of subject -2.0734 and *Lb*=0.8129*height (cm) of subject +12.328. The time interval between arm and ankle distance (*ΔTba*) was defined as the pulse transit time between brachial and tibial arterial pressure waveforms. *La* and *Lb* are estimated automatically based on subjects' height.

### Statistical analysis

Continuous variables were expressed as mean±standard deviation and categorical variables were expressed as percentages. Subjects were divided into three groups according to gender and menopausal status as follows; premenopausal and postmenopausal women, and men. Comparison of categorical and continuous variables between groups was performed using Yates' continuity corrected chi-square test and independent t-test, respectively. Relationships between baPWV and variables were assessed using linear regression analysis and Pearson's correlation coefficient. Multiple linear regression analysis was performed to examine the independent relationship between ELC and baPWV adjusting for age, BMI, WC, SBP, DBP, fasting plasma glucose, total cholesterol, HDL and LDL cholesterol, triglyceride, and smoking status. The statistical analysis was performed using SAS Ver. 9.1 (SAS Inc., Cary, NC, USA).

## RESULTS

### Characteristics of the study subjects

The study subjects stratified according to gender and menopausal status are presented in Table 1. The overall frequency of ELC was 19.02% and mean baPWV was 1,401.98±172.28 cm/s of the subjects in this study.

### Correlation between baPWV and variables examined

The correlation between baPWV and anthropometric and cardiometabolic variables are shown in Table 2. baPWV were significantly correlated with age, SBP, and DBP across three groups. However, other variables including BMI, WC, triglyceride, and HDL cholesterol were significantly correlated only in the premenopausal women group, and fasting plasma glucose was correlated only in the men and premenopausal women group. The correlation with total cholesterol and smoking status (pack per year) was not significant in any groups. The comparison of baPWV according to the presence of ELC is shown in Table 3. There was a statistically significant difference in mean baPWV with the presence of ELC in groups except for postmenopausal women (1,522.20±196.07 vs 1,426.90±171.55 cm/s, p=0.0551).

### Relationship between ELC and baPWV

Multiple linear regression analysis of all the study groups revealed that after controlling for age, BMI, WC, SBP, DBP, fasting plasma glucose, triglyceride, HDL cholesterol, LDL cholesterol, and smoking status, the presence of ELC was still independently associated with baPWV (as shown in Table 4). The significant relationship between ELC and baPWV was demonstrated regardless of gender and menopausal status.

**Figure 1:**
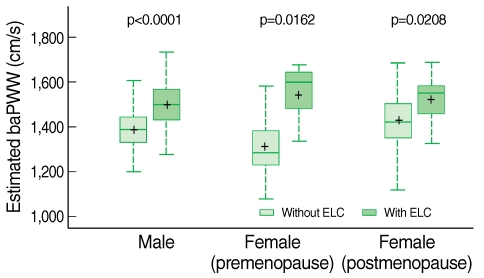
Associations between the presence of ELC and estimated baPWV in males, premenopausal females, and postmenopausal females.

Subjects with ELC had significantly higher baPWV, which estimated by the multiple regression equation, was comparable to subjects without ELC. The comparison is shown in the box plot in Figure 1.

## DISCUSSION

The current study showed that the presence of ELC was significantly associated with baPWV, independent of conventional cardiovascular risk factors in a sample of adults free of clinical event atherosclerotic diseases such as hypertension, DM and other CVD. The present study indicates that the presence of ELC demonstrated a strong association with baPWV independent of other variables and regardless of gender and menopausal status which are widely known risk factors for CVD and increased baPWV [21] in apparently healthy Korean adults.

In this investigation subjects were divided into three groups according to gender and menopausal status because previous studies had reported the influence of menopause and other conventional atherosclerotic risk factors that accelerate the progression of atherosclerosis on baPWV [22, 23]. Zaydun et al. [21] demonstrated a significant relationship between menopause and baPWV, independent of age and conventional atherosclerotic risk factors. They also suggested oestrogen deficiency may, at least in part, augment the age-related increase in arterial stiffness during the early postmenopausal phase. In this study, there were significant differences in atherosclerotic risk factors, including baPWV, between the premenopausal and postmenopausal groups (p<0.0001).

Controversy exists as to whether the presence of ELC is associated with an increased risk of CVD. However, Kaukola et al. [24] published a comprehensive investigation of the presence of ELC in CHD to provide additional support for the association. Lichstein et al. [25] showed a significant correlation between ELC and occlusion of the coronary arteries in an autopsy study. Previous researchers suggested that possible mechanisms linking ELC and CVD could be the consequences of systemic loss of elastin and elastic fibre and the aging process. However, several clinical studies demonstrated the relationship between ELC and CVD independent of age. In the current study, ELC was associated with increased baPWV independent of age, although a significant association with age was shown. baPWV is widely used in clinical studies as a surrogate marker for arterial stiffness and atherosclerotic vascular damage [16, 26]. Increased baPWV is associated with elevated levels of CHD risk factors. Currently reported data showed that baPWV may be a non-invasive risk marker of atherosclerotic CVD for the general population [19]. According to the above results and mechanisms reported in the previous studies, we may suggest loss of elastin and elastic fibre result in the development of ELC and increased arterial stiffness measured by baPWV as shown in the present study. Furthermore, the results of the present study are in accordance with are consistent with that of the recent study demonstrating ELC was significantly associated with carotid IMT [4], a marker of subclinical atherosclerosis [6, 7]. The presence of ELC, therefore, might be an independent surrogate marker of subclinical atherosclerosis in non-hypertensive and non-diabetic adults. To our knowledge, this is the first report concerning the association between ELC and baPWV in apparently healthy population.

Nonetheless, there were several limitations to our study. First, the current study had a cross-sectional design, and therefore, causal relationships could not be confirmed. A prospective study is needed to clarify the causal relationship between ELC and baPWV. Second, a standard diagnosis of atherosclerosis such as angiography and carotid-femoral PWV were not performed. Several studies suggested the usefulness of baPWV as a marker for atherosclerotic vascular damages. However, baPWV could represent peripheral arterial stiffness as well as central arterial stiffness even though subjects with self-reported peripheral arterial disease and abnormal Arterial Brachial Index (ABI) were not included in this study. Third, study subjects consisted of apparently healthy members of the Korean population with a limited sample size, and therefore, the results might not be generalisable to all ages, races or individuals with other disease status. Furthermore, the health promotion centre at which the current study was conducted is located in downtown Seoul. Subjects in this study might not be representative of the general population. Finally, the possible difference, which remains controversial, between unilateral and bilateral ELC, the depth of crease, and the length ratio of ELC to earlobe were not evaluated. Further studies that consider these limitations are needed.

In conclusion, the presence of ELC revealed a significant correlation with baPWV independent of other conventional cardiovascular risk factors in adults aged 20 yr and older. ELC is an important predictor of increased arterial stiffness measured by baPWV in asymptomatic subjects in spite of the limitations of this study. The contribution of ELC-related increase in baPWV to the prognosis of CVD should be further evaluated before these results can be extrapolated to other ethnic groups.

## Figures and Tables

**Table 1:**
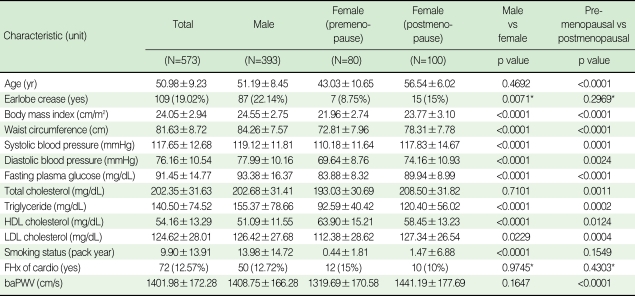
Clinical and laboratory characteristics of study subjects

Data are mean±standard deviation HDL, high-density lipoprotein; LDL, low-density lipoprotein; FHx of cardio, family history of premature coronary heart disease; baPWV, brachial-ankle pulse wave velocity. ^*^Chi-square test with Yates' continuity correction.

**Table 2:**
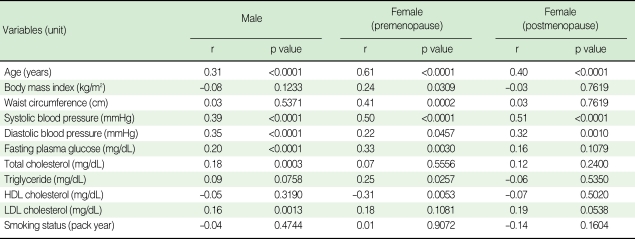
Simple correlation analysis between baPWV (cm/s) and other conventional atherosclerotic risk factors

Data are Pearson's correlation coefficient (r). HDL, high-density lipoprotein; LDL, low-density lipoprotein; baPWV, brachial-ankle pulse wave velocity.

**Table 3:**
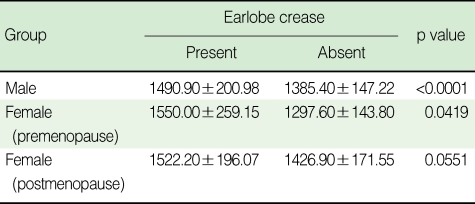
Comparisons between the presence of ELC and baPWV according to gender

Data are mean±standard deviation. baPWV, brachial-ankle pulse wave velocity.

**Table 4:**
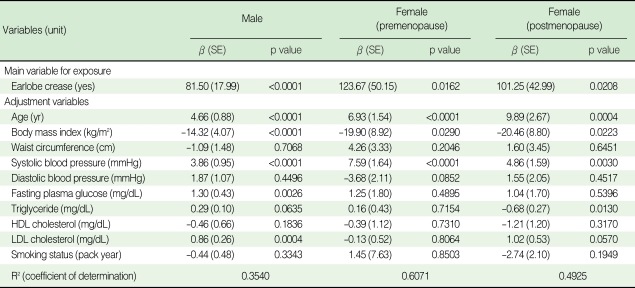
Risk factors associated with baPWV analysed by multiple linear regression analysis

baPWV, brachial-ankle pulse wave velocity; HDL, high-density lipoprotein; LDL, low-density lipoprotein; SE, standard error.
